# Predicting neurodevelopmental outcome in children born very preterm – does neonatal MRI have a role?

**DOI:** 10.1038/s41390-023-02623-0

**Published:** 2023-04-28

**Authors:** Peter J. Anderson

**Affiliations:** School of Psychological Sciences, Turner Institute for Brain & Mental Health, Monash University, Melbourne, VIC Australia

It is well established that children born very preterm (VP) are at heightened risk for an array of neurodevelopmental challenges ranging from motor and cognitive impairments to social and emotional issues,^1,2^ all of which can greatly impact an individual’s functioning in everyday life and activities. However, the severity and nature of these impairments vary from child to child, and this heterogeneity in long-term outcomes makes it challenging for health professionals to (1) provide accurate prognostic information to families, and (2) tailor support and monitoring services for individual children. Efforts to better understand the factors contributing to optimal and suboptimal outcomes in children born VP should be prioritized.

While numerous genetic, biological, social and environmental factors influence child development, we can confidently assume that neurobiological mechanisms play a crucial role given the profile of the impairments observed in children born VP.^3^ Indeed, research demonstrates that the immature brain of those born early is vulnerable to injury, which can disrupt subsequent brain maturation processes.^3^ Brain dysmaturation has been reported even in the absence of macroscopic injury,^4^ likely to be related to the non-optimal environment of the neonatal intensive care unit during one of the most critical periods of brain development.

Magnetic resonance imaging (MRI) is the most sensitive neuroimaging tool for detecting brain injury and assessing brain structure. While sophisticated post-acquisition analytic techniques are available, even for the newborn infant, these are resource intensive and available for only a select number of centers. For these reasons, there is a need for objective and reliable assessment scales of brain injury and structure that are widely accessible for clinicians and researchers, such as the Kidokoro scale.^5^ Using conventional MRI sequences, the Kidokoro assessment system assesses injury and structure of cerebral white matter, cortical gray matter, deep gray matter and cerebellum, and is used by numerous groups. Using such scales, neonatal brain injury has been shown to be associated with early developmental delay as well as poorer longer-term cognitive, motor, and behavioral functioning.^6,7^ There is also some evidence that primary brain dysmaturation, as a result of being born VP, is related to later outcomes.^8^

In this issue of *Pediatric Research*, Erdei et al.^9^ argued the need to extend existing neonatal MRI scales designed for infants born VP to include an assessment of temporal lobe structure. The temporal lobe is considered a region of particular vulnerability in infants born early, demonstrated by reduced volume, slower growth and hippocampal dysmorphology.^10–13^ The rapid growth of temporal lobe structures in the third trimester and its exposure to inflammation, sensory overload, and suboptimal nutrition during this period may explain the susceptibility of the temporal lobe. From a functional perspective, the temporal lobe is an important region as it is intimately linked to language functioning, memory and emotional status, with children born VP being at increased risk for challenges in all these domains.^14^ Given this, it is possible that a standardized assessment of temporal lobe structure in the neonatal period, may provide clinicians with additional useful information when counseling families and considering ongoing management plans for infants born VP.

With the goal of assessing temporal lobe size in infants born preterm, Erdei et al.^9^ developed a new assessment of temporal lobe size using three simple two-dimensional measurements (temporal lobe length, anterior temporal lobe extra-axial space, temporal horn width) applied to MRI scans at term equivalent age (36–42 weeks’ postmentrual age). These metrics were undertaken in 74 infants born VP and 16 infants born full-term by a combination of four raters trained to apply the measurement system. Each infant’s scan was measured by at least two raters, who were blinded to group membership. Intra- and inter-rater reliability for temporal lobe length and extra-axial space were excellent, although moderate for temporal horn width. Temporal lobe length increased by 1.2 mm per week while extra-axial space decreased by 0.4 mm per week, and as such, these measurements were corrected for postmenstrual age. The VP group had lower mean temporal lobe length, increased extra-axial space, and larger temporal horn width than the full-term group. Although trends persisted, differences in temporal lobe length and extra-axial space weakened after correcting for postmenstrual age. Temporal lobe length was positively associated with maturity and weight at birth, reduced in those with one or more neonatal comorbidities, bronchopulmonary dysplasia, and negatively related to days on parenteral nutrition. Temporal horn width was negatively associated with gestational age at birth and larger in those with cerebellar hemorrhage.

The study by Erdei et al. provides preliminary support for a new simple and objective method for assessing temporal lobe size using conventional brain MRI in neonates. Reliability was excellent except for temporal horn width, and further standardization of the procedure should be considered. The study’s sample size was modest, particularly for the full-term group, and this reduced statistical power. However, sufficient evidence was provided to suggest that this approach can be sensitive in detecting subtle differences in temporal lobe size in infants born VP, especially the highest-risk neonates such as those with bronchopulmonary dysplasia or a significant neonatal complication. As acknowledged by the authors, one of the next steps is to replicate this study with a larger cohort of VP and full-term infants, and importantly, an independent set of raters. While Erdei et al. found temporal lobe length to be associated with total brain volume, further validation is also needed by investigating the relationship between these two-dimensional metrics and temporal lobe volume. Also, given the strong correlation between temporal lobe length and total brain volume (*r* = 0.84), analyses are necessary to determine whether the temporal lobe metrics provide distinguishing information beyond overall brain size/volume. Finally, the authors provide a compelling case for focusing on the temporal lobe but applying a similar methodology to other cerebral regions is also worth considering.

Evaluating the clinical utility of these metrics is also necessary. For these temporal lobe metrics to be considered biomarkers for future development, studies are required that demonstrate an independent association with short- and long-term outcomes such as general cognitive functioning, language, episodic memory and emotional status. Assuming that future research with these metrics can confirm strong reliability, validity and clinical utility, there is potential for these temporal lobe measurements to be integrated into existing scoring systems (e.g., Kidokoro system^5^), further enhancing their applicability.

In summary, the neurodevelopmental challenges exhibited by children born VP are at least partly due to neurobiological events, and previous research clearly demonstrates that specific features on neonatal brain MRI correlate with both short- and long-term neurodevelopmental outcomes. This study by Erdei et al. extends current assessment systems by focusing on the temporal lobe, and further research will determine whether simple measurements of temporal lobe size have clinical utility. Regardless, this paper encourages a more in-depth analysis of brain injury and structure when using brain MRI with infants born VP, with the hope of ultimately improving the prediction of neurodevelopmental outcomes.
